# Efficient Oral Insulin Delivery Through Thiolated Trimethyl Chitosan-Grafted β-Cyclodextrin Nanoparticles

**DOI:** 10.3390/pharmaceutics18010097

**Published:** 2026-01-12

**Authors:** Lizhen Yu, Fengge Wang, Shuyun Bao, Yue Zhang, Xuebin Shen, Desheng Wang, Zhisheng Liu, Xinyi Liu, Lihua Li, Renmin Gong

**Affiliations:** 1 Anhui Provincial Key Laboratory for Conservation and Exploitation of Biological Resources, School of Life Science, Anhui Normal University, Wuhu 241000, China; yulizhen@wnmc.edu.cn; 2 School of Pharmacy, Wannan Medical College, Wuhu 241000, China; 20090016@wnmc.edu.cn (F.W.); shuyunbao@wnmc.edu.cn (S.B.); 20050003@wnmc.edu.cn (Y.Z.); sxbchn@wnmc.edu.cn (X.S.);

**Keywords:** oral drug delivery carrier, insulin, absorption barriers, N-trimethyl chitosan, thiolated polymer, carboxymethyl-β-cyclodextrin

## Abstract

**Background**: Oral insulin improves compliance and convenience in patients with diabetes who require regular needle injections. However, the clinical application of oral insulin preparations has been limited due to instability and inefficient permeation through the gastrointestinal tract. In this study, a novel cationic polysaccharide nanodrug delivery platform was designed for efficient oral insulin delivery. **Methods**: The innovative thiolated trimethyl chitosan-grafted β-cyclodextrin (NCT) was synthesized by utilizing N-trimethyl chitosan (TMC) as the polymer backbone. This involved modifying TMC with thiol group-containing N-acetylcysteine and carboxymethyl-β-cyclodextrin that possesses hydrophobic cavities via an amide condensation reaction. Subsequently, this polymer was employed to construct the NCT nanoparticle system using an ionic cross-linking method. The physicochemical properties of the NCT nanoparticles were systematically analyzed, and their therapeutic efficacy was comprehensively evaluated in streptozotocin (STZ)-induced animal models. **Results**: The NCT nanoparticles demonstrated mucus adhesion, permeability, and pH sensitivity, which facilitated a slow and controlled release within the gastrointestinal microenvironment due to both ionic electrostatic interactions and disulfide bonding interactions. The experiments revealed in vivo that insulin/NCT nanoparticles extended the retention time of insulin in the small intestine. Blood glucose levels decreased to approximately 39% of the initial level at 5 h post-administration while exhibiting smooth hypoglycemic efficacy. Simultaneously, insulin bioavailability increased to 12.58%. **Conclusions**: The NCT nanoparticles effectively protect insulin from degradation in the gastrointestinal microenvironment while overcoming intestinal barriers, thereby providing a promising approach to oral biomolecule delivery.

## 1. Introduction

Diabetes mellitus (DM), which is a metabolic endocrine disease characterized by chronic hyperglycemia, is one of the most prevalent chronic metabolic diseases worldwide [1,2]. Patients with type 1 diabetes mellitus (T1DM) typically require multiple daily injections of rapid-acting insulin and one or two injections of long-acting insulin or continuous insulin infusion via an insulin pump to maintain normal blood glucose levels [3]. However, subcutaneous and transdermal injections present challenges such as inconvenient administration and dose limitations [4]. Additionally, the injection mode of drug delivery could cause several pathological conditions, including hypoglycemia, lipoatrophy, hypertrophy at the injection site, local allergic reactions, erythema, itching, abscesses, and hard nodules, which lead to poor patient compliance [5,6].

Currently, first-line clinical drugs for DM can be categorized into insulin and non-insulin drugs [7]. Insulin drugs include rapid-acting (e.g., lysergic acid and glutaraldehyde insulin), short-acting (e.g., regular human insulin), and long-acting (e.g., glycemic acid insulin) insulin preparations [8,9]. Conversely, non-insulin drugs primarily comprise chemical agents such as biguanides, α-glucosidase inhibitors, and sulfonylureas, as well as protein–peptide hypoglycemic agents such as glucagon-like peptide (GLP-1) receptor agonists [7,10]. Despite the development of non-insulin-based drugs with novel targets and mechanisms, insulin-based treatments remain critical due to their high selectivity and efficacy [5,11,12]. Especially in T1DM management, multiple subcutaneous injections of exogenous insulin are the primary means of controlling blood glucose. However, they often lead to poor glucose regulation since insulin bypasses the liver and directly enters systemic circulation.

Improving DM therapy delivery strategies, including oral [13,14], intranasal [15], pulmonary [16], and transdermal routes [17], is becoming increasingly sophisticated. Oral insulin delivery, in particular, reduces pain and inflammation associated with subcutaneous injections and improves portal blood medication levels while decreasing the incidence of peripheral hyperinsulinemia [18]. Although oral insulin is susceptible to hepatic first-pass metabolism, reducing its overall bioavailability lowers the risk of hypoglycemia and peripheral tissue immune responses that occur with parenteral administration [18]. This method enhances patient compliance, reduces side effects, and alleviates complications in DM patients [19].

However, oral insulin exhibits a bioavailability of less than 2% due to its high molecular weight, hydrophilicity, poor stability, and low tolerance to protease hydrolysis, complicating oral insulin development [20]. The pharmacokinetic and pharmacodynamic characteristics of oral insulin preparations vary based on the absorption site (gastrointestinal tract) and route (cellular transmembrane transport or paracellular pathway). Rapid absorption occurs via the paracellular pathway [21], while slow absorption occurs via the cellular pathway (through intestinal epithelial cells or M-cell endocytosis) [22]. Insulin absorption in the gastrointestinal tract is hindered by multiple physical and biochemical barriers, including low pH, enzymatic degradation, mucus barriers, intestinal epithelial cells, and efflux pumps, which significantly limits insulin uptake [23,24]. Researchers are exploring various strategies to facilitate oral insulin delivery, including the use of osmotic enhancers, enzyme inhibitors, nanoparticle carriers, cell-penetrating peptides, intestinal patches, and microneedle devices [23]. With the advancement of nanotechnology, various oral insulin nanodrug delivery systems have been developed, including liposomes, metal ions, proteins, amino acids, and natural and synthetic polymers [25]. These systems prolong the residence time of the drug at the intestinal absorption site, improving drug penetration and bioavailability [20,26,27].

To overcome the multiple absorption barriers of oral insulin, the present study successfully constructed a novel thiolated chitosan nanoparticle, emphasizing the significant impact of small adjustments to surface chemistry on bioavailability. In this study, we developed a novel thiolated trimethyl chitosan-grafted β-cyclodextrin (NCT) by utilizing N-trimethyl chitosan (TMC) as the polymer backbone, modifying it with thiol group-containing N-acetylcysteine (NAC) and carboxymethyl-β-cyclodextrin sodium salt (CMCD) with hydrophobic cavities via the amide condensation reaction. NCT nanoparticles (NCT NPs) demonstrated mucus adhesion, permeability, and pH sensitivity, providing slow and controlled release in the gastrointestinal microenvironment due to ionic electrostatic and disulfide bonding interactions. The experiments showed in vivo that insulin-loaded NCT NPs (insulin/NCT NPs) extended insulin retention time in the small intestine. Blood glucose levels decreased to approximately 39% of the initial level at 5 h post-administration, with the hypoglycemic effect sustained for nearly 6 h, demonstrating smooth and sustained efficacy. Simultaneously, insulin bioavailability increased to 12.58%, 18-fold higher than that of the free insulin oral control group. A safety assessment study confirmed that NCT NPs are safe oral protein–peptide drug delivery vehicles. Collectively, NCT NPs could serve as potential oral nanodelivery vehicles for protein and peptide drugs.

## 2. Materials and Methods

### 2.1. Materials

Chitosan (CS) with 40 kDa MW and 90% deacetylation was bought from Sangon Biotech (Shanghai, China). Carboxymethyl-β-cyclodextrin sodium salt (CMCD, MW: 1591) with a purity level of 98% was purchased from Binzhou Zhiyuan Biotech (Shanghai, China). N-acetylcysteine (NAC) was acquired from Aladdin Industrial Corporation (Shanghai, China). Insulin (28 IU/mg) was purchased from Wanbang Jinqiao Pharmaceutical Co. Ltd. (Xuzhou, China). N-hydroxysuccinimide (NHS), 1-ethyl-3-(3-dimethylaminopropyl) carbodiimide (EDC), sodium tripolyphosphate (TPP), and streptozotocin (STZ) were obtained from Sigma-Aldrich (St. Louis, MO, USA). Cy5.5-NHS, fluorescein isothiocyanate (FITC), and DAPI solutions were purchased from Dalian Meilun Biotechnology Co., Ltd. (Dalian, China). All the reagents and chemicals used in this study were of analytical grade.

### 2.2. Cells and Animals

The Caco-2 cell line, consisting of a type of human epithelial colorectal adenocarcinoma cells, was sourced from the Cell Bank of the Chinese Academy of Sciences (Shanghai, China). Additionally, HT29-MTX-E12 (E12) cells, which could secrete abundant adherent mucus [28], were purchased from the European Collection of Authenticated Cell Cultures (ECACC, Salisbury, UK).

Adult male ICR mice, weighing 20 ± 2 g, were acquired from the Qinglong mountain animal breeding ground (Nanjing, China) and were raised in an SPF environment in the Animal Laboratory Centre of Wannan Medical College with a temperature of 23 ± 2 °C and relative humidity of 55 ± 10%, under 12 h light–dark cycles, and they freely accessed food and water for one week before the experiment. All experimental procedures involving these animals were performed in compliance with protocols approved by Experimental Animal Welfare and Ethics Committee of Wannan Medical College (WNMC-AWE-2024112). All surgical procedures were performed under anesthesia via an intraperitoneal injection of 1% pentobarbital sodium at 50 mg/kg, subsequently subjected to humane sacrifice through cervical dislocation once the following endpoints were reached: rapid hypothermia and respiratory impairment. Death was confirmed by the sustained absence of cardiac activity and pupillary dilation for a 5 min observation period.

### 2.3. Synthesis of NCT Polymers

In this study, TMC was synthesized via a one-step methylation method, leveraging the reactivity of the amino groups at the C2 position of CS as previously described [29]. Specifically, 2.0 g of CS was dispersed in 50 mL of N-methylpyrrolidone (NMP) and magnetically stirred at room temperature for 2 h to ensure complete swelling. Subsequently, 5 mL of 20% (*w*/*v*) sodium hydroxide solution, 10 g of sodium iodide, and 12 mL of methyl iodide were added sequentially. The reaction system was heated to 60 °C and stirred at a speed of 400 r/min for 12 h. Upon the completion of the reaction, the product was subjected to ethanol precipitation, diethyl ether washing, deiodination in sodium chloride solution, and dialysis in a dialysis bag (MW 8–10 kDa). The dialyzed product was freeze-dried under vacuum (−50 °C, 1 Pa) to a constant weight and then stored at 4 °C.

**Figure 1:**
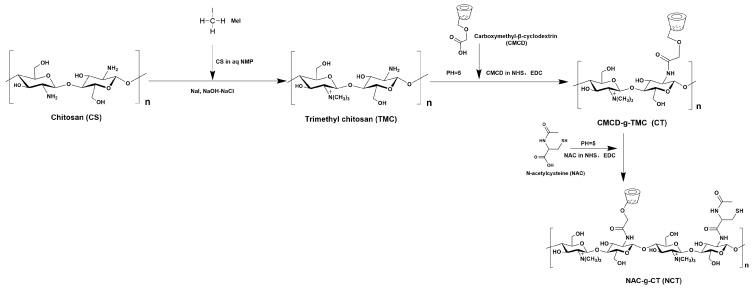
Synthetic schematic diagram of CT and NCT.

Subsequently, amide bond formation occurred between the residual amino groups of TMC and the carboxyl groups of NAC and CMCD through the mediation of EDC/NHS, resulting in the modification of TMC with NAC and CMCD (Figure 1). Briefly, EDC/NHS/NAC and EDC/NHS/CMCD (2:2:1 molar ratio) were dissolved in distilled water under continuous magnetic stirring (600 rpm) for 2 h. Subsequently, the CMCD and NAC activation solutions were sequentially infused dropwise into a double-volume TMC solution (5 mg/mL) at 1 h intervals under constant stirring. Following this, the mixture pH was adjusted to 5.0 with HCl/NaOH and maintained (±0.1), with a subsequent reaction at 25 °C for 12 h under light-protected conditions. Then, the NCT product underwent dialysis at 4 °C with pH 5.0 (MW 3500 Da) for 5 d to remove the remaining reagents. Eventually, the polymer solutions were subjected to lyophilization and then stored at 4 °C for future use.

### 2.4. Physicochemical Characterization of NCT Polymers

To confirm the successful synthesis of NCT polymers, the molecular structure was characterized by Fourier transform spectroscopy (FTIR) and ^1^H NMR. The FTIR spectra of NCT, NAC, CMCD, and TMC were measured using an FTIR spectrometer (Nicolet iS5, Thermo Fisher Scientific, Waltham, MA, USA), and this involved combining the samples with KBr, compacting them into pellets, and subsequently examining them within the spectrum ranging from 4000 to 400 cm^−1^. To acquire ^1^H NMR spectra, an appropriate amount of the sample was dissolved in D_2_O and analyzed using a 400 MHz NMR spectrometer (AV-400, Bruker, Fällanden, Switzerland).

The extent of N-trimethyl group incorporations considered an index of the degree of quaternization (DQ) in TMC was determined by ^1^H NMR using the following equation [30]:

$$DQ\;\left(\%\right)\;=\;\frac{Integral\;of\;[\;{N\left({CH}_{3}\right)}_{3}]}{Integral\;of\;[H2,H3,H4,H5,H6]}\times \frac{6}{9}\times 100$$

### 2.5. Fabrication of NCT NPs

The NCT-based NP series was prepared using ion cross-linking and TPP as the cross-linker [31]. Blank NCT NPs (NCT NPs) were formulated by gradually dropping TPP solution into NCT solution with continuous stirring. Briefly, NCT and TPP polymers were separately dissolved in deionized water and sonicated for 5 min at ambient temperature. Subsequently, the solutions were individually filtered through a 0.45 μm membrane to obtain 2 mg/mL NCT solution and 2 mg/mL TPP solution. Subsequently, an appropriate amount of TPP solution was slowly added to a 5 mL NCT solution under continuous stirring at 1000 rpm for 4 h, resulting in the formation of an opalescent suspension. Subsequently, the NP suspension underwent filtration by the 0.45 μm membrane to eliminate dust and impurities to obtain NCT NPs for the subsequent characterization. After centrifugation and vacuum freeze-drying, NCT NPs were obtained as a powder and stored at 4 °C for subsequent experiments. Blank CT NPs (CT NPs) were fabricated by the same preparation procedure.

Following a similar procedure, insulin-loaded NCT NPs (insulin/NCT NPs) were fabricated by encapsulating a specific amount of insulin prior to the initiation of the gelation reaction. Initially, insulin was fully dissolved in a hydrochloric acid solution (0.01 M) to obtain 1 mg/mL insulin solution. Based on the different weight ratios of insulin to NCT (1:20–1:50), the insulin solutions were dropped into the NCT solution (2 mg/mL) and magnetically agitated for 12 h against exposure to light. Following this step, an appropriate amount of TPP solution was added dropwise at a controlled rate (0.5 mL/min) to the stirring insulin–NCT mixture, with subsequent continuous agitation for 4 h. The centrifugation supernatant was collected and subjected to measure the encapsulation efficiency (EE) and drug loading content (LC). The sediment was freeze-dried to obtain insulin/NCT NP powder. Insulin-loaded CT NPs (insulin/CT NPs) were obtained via an identical preparation protocol.

### 2.6. Characterization of NCT Nanoparticles

The NPs were analyzed for particle size (z-average), polydispersity index (PDI), and zeta potential through dynamic light scattering at 25 °C with a scatting angle of 90° using a Zetasizer Nano ZS90 (Malvern, UK). Furthermore, the particle morphology was observed using transmission electron microscopy (TEM) (JEM-2100F, Tokyo, Japan). The EE and LC of insulin in the insulin/NCT NPs were measured using HPLC [32]. The EE and LC were calculated using the following mathematical formulas:

$$EE\;(\%)=\frac{M_{total\;ins}-M_{free\;ins}}{M_{total\;ins}}\times 100$$

$$LC\;(\%)=\frac{M_{total\;ins}-M_{free\;ins}}{M_{total\;NPs}}\times 100$$

where M_total ins_ represents the total quantity of insulin added, M_free ins_ is the quantity of free insulin in the supernatant, and M_total NPs_ is the total quantity of nanoparticles.

An insulin release evaluation of NCT NPs was performed in simulated gastric fluid (SGF, pH 1.2) and simulated intestinal fluid (SIF, pH 7.4) [32]. Briefly, 5 mL of insulin/NCT NPs was introduced into a dialysis bag with a molecular cutoff ranging from 8000 to 14,000 Da. Following this, the dialysis bag was placed in 10 mL of SGF at 37 °C and gently swayed at 80 rpm. After 2 h, the dialysis bag was transferred to an equal volume of SIF and shaken continuously at the same speed for 6 h. At predetermined time points, 0.2 mL of the release medium (SGF or SIF) was removed and replaced with an equal volume of the artificial simulation liquid. The amount of insulin released was directly quantified using HPLC.

The mucoadhesion and penetration of insulin/NCT NPs through the mucus layer were evaluated according to a previously reported method [33,34]. Briefly, insulin was initially FITC-labelled as described previously [32]. Porcine intestinal mucus was harvested and purified according to the standard protocol [35]. Subsequently, 500 μL of FITC–insulin/NCT NPs or FITC–insulin/CT NPs was homogenized with 200 mg mucus and incubated (37 °C, 80 rpm). At 15 and 30 min, samples were centrifuged at 2000 rpm for 10 min, and the supernatant’s fluorescence intensity (FI) was determined via fluorescence spectrophotometry (Ex: 485 nm; Em: 525 nm), and the adhesion rate was calculated as follows: Adhesion rate (%) = (1 − FI_s_/FI_bc_) × 100% (FI_s_: sample fluorescence intensity; FI_bc_: blank control fluorescence intensity).

### 2.7. The Retention Assay In Vivo

The gastrointestinal persistence of insulin/NCT NPs was monitored using In Vivo Imaging System (IVIS). First, Cy5.5–insulin was synthesized by adding 400 μL Cy5.5-NHS (0.25 mg/mL) to 10 mL insulin (4 mg/mL), and the mixture was stirred in darkness at 4 °C for 12 h. Unconjugated Cy5.5-NHS and other small molecules were removed by dialysis until the dye was no longer detectable. Subsequently, the Cy5.5–insulin solution was lyophilized at −40 °C and stored desiccated at −20 °C for subsequent studies. The mice were fasted for 12 h with ad libitum access to water prior to experimentation.

The formulated Cy5.5–insulin/NCT NPs were orally administered to mice at an insulin-equivalent dose of 50 IU/kg. Subsequently, the mice were sacrificed at 0.5 h, 2.0 h, 4.0 h, and 6.0 h post-administration. Immediately, gastrointestinal tracts (stomach, duodenum, jejunum, ileum) were removed for fluorescence distribution and intensity analysis under EX 695 nm excitation and EM 707 nm emission.

The fluorescence intensity of Cy5.5–insulin in various gastrointestinal segments was determined using Living Image software (version 4.5.2, PerkinElmer, Springfield, IL, USA).

### 2.8. Hypoglycemic Effect and Pharmacokinetic Behaviour in Mice with DM

The hypoglycemic efficacy of orally administered nanoparticles and free insulin was evaluated in STZ-induced diabetic mice relative to subcutaneously injected insulin solution. To induce DM, male mice were intraperitoneally injected with STZ solution (50 mM citrate buffer solution, pH 4.5) at a dose of 200 mg/kg, as previously reported [36]. After one week, mice with blood glucose levels of 16.5–28.5 mM were considered mouse models of DM. All diabetic mice were divided randomly into three experimental groups, followed by the administration of oral insulin/NCT NPs (50 IU/kg) or oral free insulin (50 IU/kg) or subcutaneous injection of free insulin solution (5 IU/kg, positive control). Blood samples were collected from the tail vein before drug administration and at each time point after dosing. Blood glucose levels were measured using a glucose meter (GA-3, Sinocare, Changsha, China). Plasma insulin levels were quantified using mouse insulin ELISA immunosorbent assay (ABclonal Biotechnology Co., Ltd., Woburn, MA, USA). The bioavailability (F) relative to subcutaneous injection was determined with the following formula:

F (%) = (AUC (oral) × Dose (s.c.))/(AUC (s.c.) × Dose (oral)) × 100

where AUC is the area under the plasma insulin concentration curve, and s.c. denotes subcutaneous injection.

### 2.9. Statistical Analysis

All assays included at least 3 biologically independent replicates. Data are reported as the mean ± SD. Intergroup differences were statistically analyzed using Student’s *t*-test (two-tailed, *p* < 0.05 significant).

## 3. Results and Discussion

### 3.1. Preparation and Characterization of NCT

**Figure 2:**
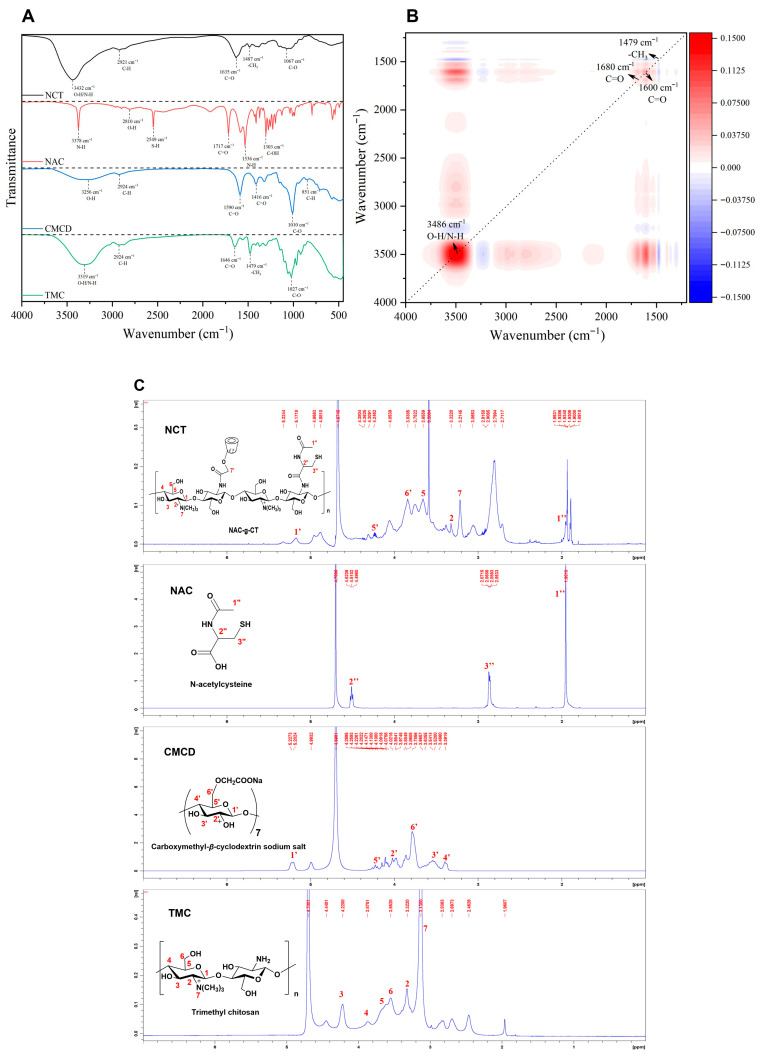
(**A**) FTIR spectra of NCT, NAC, CMCD, and TMC. (**B**) 2DCoS of NCT vs. TMC: red denotes positive correlation, and blue denotes negative correlation. (**C**) ^1^H-NMR spectra of NCT, NAC, CMCD, and TMC.

In this study, a novel polymeric backbone was constructed for nanoloaded drugs by grafting two small molecules, NAC and CMCD, onto the long chain of TMC using CS as the backbone, primarily through amidation reactions. The methylation of TMC was confirmed by FTIR analysis (-CH_3_ at 1479 cm^−1^) and ^1^H NMR (δ = 3.15 ppm, 3H), as shown in Figure 2C. As a backbone molecule, TMC plays a pivotal role in subsequent studies, and its inherent properties, especially the degree of quaternization (DQ), were of particular significance. Accordingly, we successfully synthesized quaternized chitosan (TMC), which was found to have an approximate DQ of 33%. This modification not only effectively enhanced the water solubility and cationic properties of chitosan but also preserved ample reactive amino groups on the sugar rings of TMC for subsequent grafting and coupling reactions.

The infrared characteristics of NCT, NAC, CMCD, and TMC are presented in Figure 2A. The infrared features of each substrate were relatively distinct. Specifically, the absorption peaks of NAC were well-resolved. The sharp peak at 3378 cm^−1^ was mainly attributed to the N-H stretching vibration mode. The absorption peak at 2810 cm^−1^ was a typical characteristic of the carboxylic acid dimer O-H structure. Owing to intermolecular hydrogen bonding interactions, broad peaks usually appear in this region, which also serves as crucial evidence for the existence of free carboxyl groups. The FTIR spectrum of CMCD exhibits the typical characteristics of carboxymethyl-β-cyclodextrin (sodium salt): the absorption peak at 1590 cm^−1^ was assigned to the asymmetric stretching vibration of coordinated carboxyl C=O, while that at 1416 cm^−1^ corresponded to the symmetric stretching vibration of coordinated carboxyl C=O [37,38]. Similarly, in TMC’s infrared spectrum, the absorption peak at 1479 cm^−1^ was ascribed to the methyl bending vibration mode of the quaternary ammonium group, representing the material’s most prominent characteristic peak [39,40]. Following polymerization, the spectral characteristics of NCT were highly similar to those of precursor TMC, while sharp fine absorption bands were lost and replaced predominantly by broad absorption peaks. This phenomenon stemmed from the formation of a composite with strong intermolecular interactions by NAC, CMCD, and TMC during the polymerization process, which resulted in restricted molecular motion and reduced homogeneity of the chemical microenvironment, thus imparting the overall spectral bands with broadening characteristics consistent with amorphous polymers. The elevated relative intensity and stretched peak shape of the O-H and N-H absorption peaks around 3432 cm^−1^ also confirmed that the polymerization process greatly enriched and complicated the hydrogen bond network within the system.

To further characterize the subtle structural differences between NCT and its substrate TMC, we compiled a dataset with the infrared spectra of these two samples and performed two-dimensional correlation spectroscopy (2DCoS) analysis (Figure 2B) [41]. As observed in the original spectra, remarkably strong signal changes corresponding to O-H and N-H stretching vibrations were detected near 3486 cm^−1^, and identical signal changes were also present near 1680 cm^−1^ and 1600 cm^−1^. On the one hand, the appearance of these two signals is associated with the introduction of C=O structures from NAC and CMCD; on the other hand, it may be caused by the condensation reaction between the carboxyl groups of NAC and the amino N-H groups of TMC or CMCD, which generates amide linkages. Moreover, electrostatic interactions also take place between the quaternary ammonium cations (-N^+^(CH_3_)_3_) of TMC and the carboxylate anions of CMCD, leading to the formation of ionic-type complexes. The presence of a negative correlation signal around 1479 cm^−1^ further corroborates that the chemical microenvironment of TMC’s quaternary ammonium cations experienced remarkable changes during the polymerization process, and their relative intensity was notably reduced owing to intense perturbation or restriction by the carboxylate anions of CMCD. The aforementioned characteristics confirm that NAC, CMCD, and TMC successfully constructed composite NCT through the synergistic actions of amidation, electrostatic complexation, and other interfacial mechanisms.

Additionally, as shown in Figure 2C, the ^1^H NMR spectrum of NCT reproduced the characteristic proton vibrational peak bands of the constituent molecules after covalent grafting. However, the chemical shifts in the protons of the constituent residues showed mild red or blue shifts owing to the electronegativity of the constituent groups of NCT and the effects of hydrogen bonding and decoupling within the NCT molecule. Specifically, the ^1^H NMR spectrum of NCT in the region of 3.30 to 4.38 ppm exhibited a band of NMR peaks for protons, which were fundamental structural units in the molecular skeleton of TMC polysaccharides. The -N^+^(CH_3_)_3_ protons at 3.21 ppm indicated a mild red shift to the high field, which increased the density of the electron cloud by absorbing free electrons from the surrounding atoms of the moiety, resulting in a mild change in the chemical shifts. The overall NCT preserved the basic peak spectral features of the TMC molecular skeleton, with a series of proton magnetic spectral peaks of the cavity ligand CMCD simultaneously presented in the NCT in the 3.56–5.16 ppm region. Although a partial overlap with the TMC proton peak was observed, some of the peak shapes of the CMCD residuals were still maintained at 3.56–5.16 ppm, indicating that CMCD had been successfully introduced into the TMC molecular chain.

Collectively, in the hydrogen spectrum of NCT, the hydrogen signals of H-2, 5, and 7 in the raw material TMC were observed, indicating that NCT contained TMC. The appearance of H-1,6 signals indicated that NCT was grafted with a CMCD portion, and the appearance of the methyl hydrogen signal of H-1 indicated that NAC grafting was successful. The coupling efficiency of graft polymerization of CMCD was approximately 70.32 ± 1.68%, and the mass percentage of CMCD in NCT was 14.06 ± 0.34%. Additionally, the thiol content of the polymers was determined using Ellman’s reagent [42]. Spectrophotometric analysis using Ellman’s reagent confirmed the presence of thiol groups in the polymer backbone. The thiol content of NCT was 126.94 ± 2.62 μg/g, and the presence of thiol groups confirmed the success of thiolation.

### 3.2. Preparation and Characterization of NCT NPs

**Figure 3:**
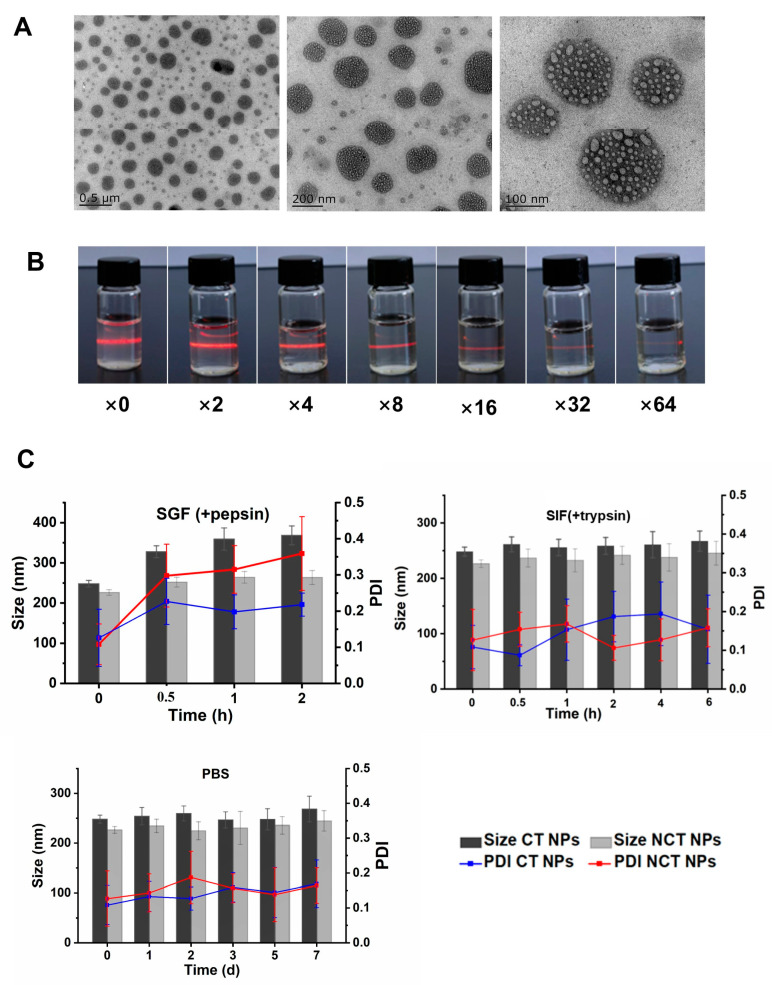
The characterization of NCT NPs. (**A**) TEM micrographs of NCT nanoparticles. (**B**) The Tyndall phenomenon of NCT NPs under different dilution multiples of PBS. (**C**) The changes in the particle size and PDI of nanoparticles in different pH environments: simulated gastric fluid (SGF) containing pepsin, simulated intestinal fluid (SIF) containing trypsin, and PBS.

The ionic cross-linking method was used to prepare the nanoparticles because the P_3_O_10_^5–^ contained in the TPP solution was primarily neutralized with -N(CH_3_)_3_^+^ in NCT, as well as monomethylated sites (-N(CH_3_)H_2_^+^), bimethyldimethylated sites (-N(CH_3_)_2_H^+^), and even with purely protonated amines (-NH_3_^+^). The pKa values of mono- and bimethylamines are approximately 6, which means that all these amine groups are ionized and interact with TPP when the pH of the nanoparticle suspension is below 6. As shown in Table S1, the average particle size of NCT NPs was 224.86 ± 7.22 nm with a zeta potential of +(25.5 ± 3.90) mV, whereas the average particle size of CT NPs was 242.45 ± 7.32 nm with a zeta potential of +(26.8 ± 4.09) mV. The two types of nanoparticles were similar in terms of particle size and zeta potential. The PDI was <0.3, indicating that the two nanoparticle systems were relatively stable and that the particle size distribution was uniform. According to our experimental test results, the zeta potential of both nanoparticles was positive, but with an increase in polymer substitution, the zeta potential slightly decreased. The average particle size of the two nanoparticles was in the range of 220–240 nm, and the combined absolute zeta and PDI values proved that the system of the prepared NPs was stable. As shown in Figure 3A, the NCT NPs were mostly spherical or nearly spherical, with a particle size of approximately 200 nm. The NCT NPs were individually dispersed as spherical particles in low-magnification TEM images. In the high field of view, the surface boundary of the nanoparticles was clear and smooth, and the nanoparticles exhibited a fibre-like mesh, which could be attributed to the cross- linking of the polysaccharide chains of the NCT skeleton. As shown in Figure 3A, the particle sizes of the NCT NPs measured using TEM were slightly smaller than the average particle sizes measured using dynamic light scattering (DLS). This was because the nanoparticles used for TEM were dried nanoparticles. In contrast, the nanoparticles used for DLS are NCT NPs in the aqueous dispersion system, which had a hydration layer due to the hydration on the surface of the nanoparticles. After drying, the NCT NPs lost their surface hydration structure, and thus, the particle sizes were slightly smaller. Simultaneously, a small number of tiny particles appeared in the field of view after TEM observation, which may be due to the existence of a trace amount of the NCT self-assembly phenomenon during the nano-preparation process and the emergence of tiny “shell–core” vesicle structure.

Moreover, the effect of different dilution times on the NCT properties was explored. As shown in Figure 3B, the dilution of the Tyndall phenomenon decreased with increasing dilution, and the appearance of the solution gradually faded from a transparent pale blue milky liquid to a colourless transparent solution, with an apparent change in particle size and PDI. Therefore, it was speculated that the properties of the nanoparticles did not change significantly after NCT was orally ingested and diluted in large quantities. However, the pH of the gastrointestinal tract is believed to be one of the main factors limiting the oral absorption of protein peptides. Since the pH in the stomach ranges from 1.0 to 2.5, increases to 6.6–7.5 from the proximal small intestine to the ileum, and subsequently decreases to 6.4 in the cecum, this pH variation in the gastrointestinal tract makes it difficult for the preparation of nanoparticles to maintain nanoparticle stability throughout the gastrointestinal tract. Therefore, preparing pH-sensitive oral drug delivery systems is crucial for overcoming the effects of the strongly acidic stomach environment on protein peptides.

As shown in Figure 3C, the stability of NCT NPs in solution was evaluated by measuring the particle size distribution and polydispersity index (PDI) in the media. The experimental results showed that the average particle size and PDI of NCT NPs did not change significantly with time in either simulated gastric fluid (SGF) or simulated intestinal fluid (SIF) solutions. The average particle size changed slightly when placed in PBS for 7 d. The experimental results showed that the NCT NPs exhibited good stability in the three solution media. The stability of the NCT NPs was related to the formation of disulfide bonds in the nanoparticles, with a presence that enhanced their stability. Additionally, NCT NPs could be stabilized through the hydrophobic effect of the hydrophobic cavities.

### 3.3. Drug Release In Vitro

**Figure 4:**
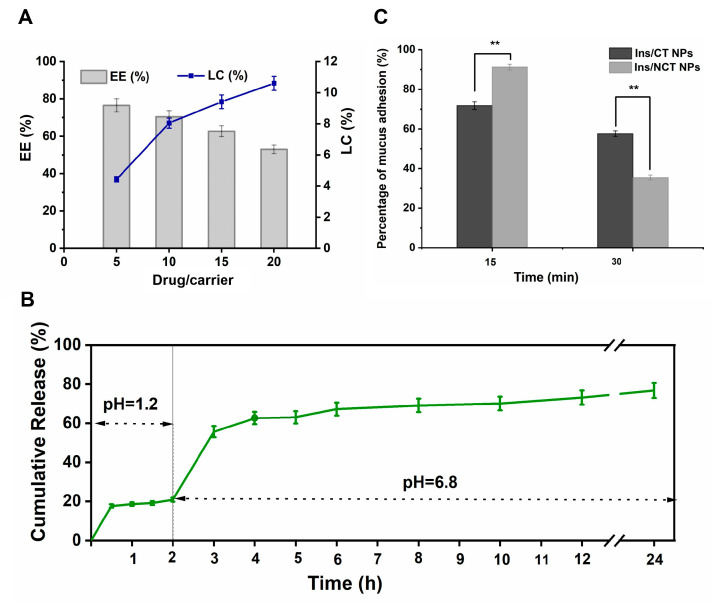
The characterization of insulin/NCT NPs. (**A**) The encapsulation and loading rates of insulin/NCT NPs. (**B**) The cumulative release profiles of insulin from NCT nanoparticles in simulated gastric medium with pH 1.2 and in simulated intestinal medium with pH 6.8 in vitro. (**C**) The mucus adhesion rates of different nanoparticles. All values are presented as means ± SD (*n* = 3). ** *p* < 0.01.

As oral delivery carriers of insulin, NCT NPs should have favourable encapsulation efficiency (EE) and loading capacity (LC) properties, which helped reduce the production cost of the oral formulation of insulin/NCT NPs, as well as ensuring an effective therapeutic dosage. In this study, the drug–carrier mass ratio was used as an optimization factor to study the EE and LC of insulin at different ratios. The insulin content was determined, and EE and LC were calculated using HPLC. As shown in Figure 4A, the encapsulation rate of insulin gradually decreased with an increasing drug–carrier mass ratio in the range of 5/100–20/100, whereas the drug loading capacity showed an increasing trend. When the drug–carrier ratio was 10/100, the encapsulation rate was 70.42 ± 1.98%, and the drug loading capacity was 8.04 ± 0.32%, thus exhibiting more desirable drug encapsulation characteristics.

Insulin was readily degraded by gastrointestinal enzymes, so slowing the rate of insulin release from the formulation to provide a concentration gradient for intestinal absorption would be a favourable factor. As shown in Figure 4B, the insulin NCT NPs showed continuous release in both SGF and SIF media. In SGF, only a small amount of insulin was released, and subsequently, in SIF, insulin release showed a substantial increase. Furthermore, after 4 h, insulin could be released slowly and continuously in SIF, and by 24 h, the cumulative release rate had increased to 76.46 ± 3.86%. This characteristic of insulin released in the gastrointestinal environment was related to the formation of disulfide bonds in the NPs complexed with hydrophobic cavities. Collectively, NCT NPs as insulin oral delivery carriers could effectively protect insulin transport to the absorption site and enabled slow and continuous release, presenting desirable gastrointestinal release properties.

Additionally, the enhanced wall adhesion properties contributed to increased drug absorption by inducing the encapsulated drug to remain at the absorption site for a longer time [43,44,45]. However, particles loosely attached to the outer mucus layer are easily removed because of the rapid turnover of mucus cells. This rapid removal was the main factor that complicated oral drug delivery. Therefore, ensuring that nanoparticles adhered to the intestinal wall and then penetrated the mucus to reach the top of the intestinal epithelium, thus improving cellular uptake, is the challenge and focus of current research on oral nanodelivery systems [33,46,47]. Mucin adhesion experiments showed that insulin/NCT NPs exhibited stronger mucin adhesion and penetration than insulin/CT NPs. As shown in Figure 4C, after 15 min of incubation with mucin, the adhesion rate of insulin/NCT NPs was as high as 91%, which was significantly enhanced compared with that of the insulin/CT NP group (*p* < 0.01). After 30 min, the adhesion rate of both groups of NPs decreased, and that of insulin/NCT NPs decreased to 35%, which was greatly lower than that of the insulin/CT NP group (*p* < 0.01). Notably, the negatively charged mucins on the surface could adsorb insulin/NCT NPs through hydrophobic or electrostatic interactions, which caused the NPs to stay in the mucus layer. The disulfide exchange reaction or disulfide bond formation between the thiol groups on insulin/NCT NPs and those on mucus further improved mucus adhesion. In insulin/NCT NPs adhering to the mucus layer, mucus adhesion was reduced owing to the residual thiol groups that could lead to partial disulfide bond (-S-S-) breakage in the mucin and thereby promote the mucus penetration of NPs.

### 3.4. Cellular Uptake Studies

**Figure 5:**
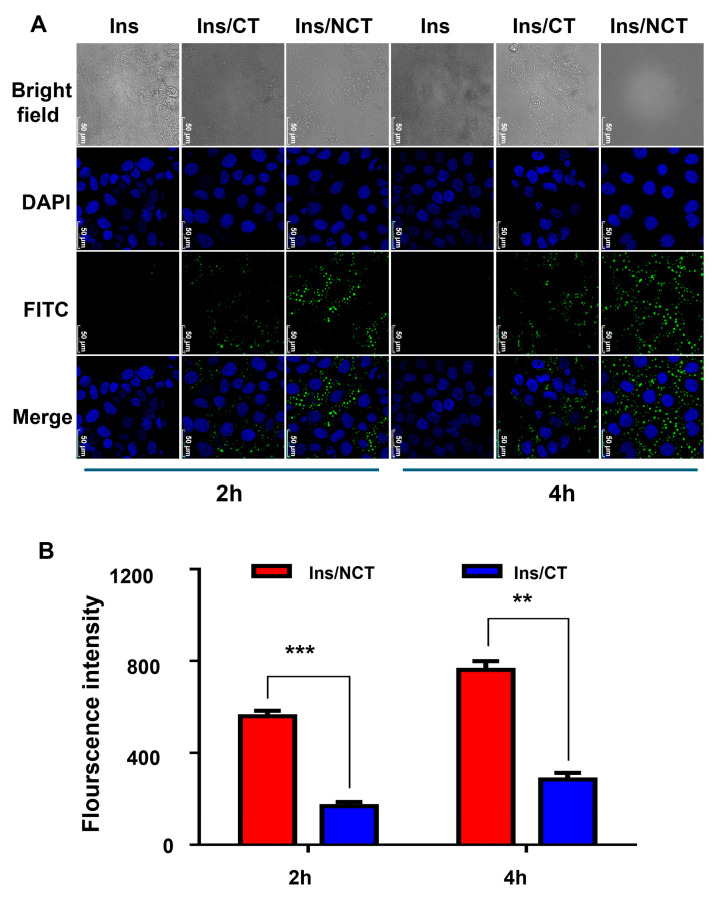
(**A**) Confocal micrographs of different formulations of Caco-2/E12 cell monolayers for 2 h or 4 h. Cell nuclei were stained with DAPI. Scale bar, 50 μm. (**B**) Quantitative fluorescence analysis of cell uptake by Caco-2 cells. Total intensity of green fluorescence in each group of CLSM images was analyzed and calculated through Image J software(version 1.53e, National Institutes of Health, Bethesda, MD, USA). All values are presented as means ± SD (*n* = 3). ** *p* < 0.01, *** *p* < 0.001.

In this study, Caco-2 cell/E12 cell co-cultures were used to simulate the uptake of small intestinal cells in vitro. As shown in Figure 5A, more notable FITC fluorescence signals around the nucleus were observed, which proved that FITC–insulin/NCT NPs could be taken up by Caco-2 cells in the co-culture model in the 2 h and 4 h groups, and the FITC–insulin/NCT NP group had stronger green fluorescence signals than the FITC–insulin/CT NP group. As shown in Figure 5B, the signals and fluorescence intensities of the two groups showed significant differences at 2 h and 4 h, indicating that the FITC–insulin/NCT NPs had a better epithelial cell transporter ability. The FITC fluorescence signal observed in the FITC–insulin/CT NP administration group was relatively weak because of the mucus barrier formed by mucin secretion from E12 cells. This suggests that the NCT NPs offer an advantage over the CT NPs in terms of mucus penetration and cellular uptake.

**Figure 6:**
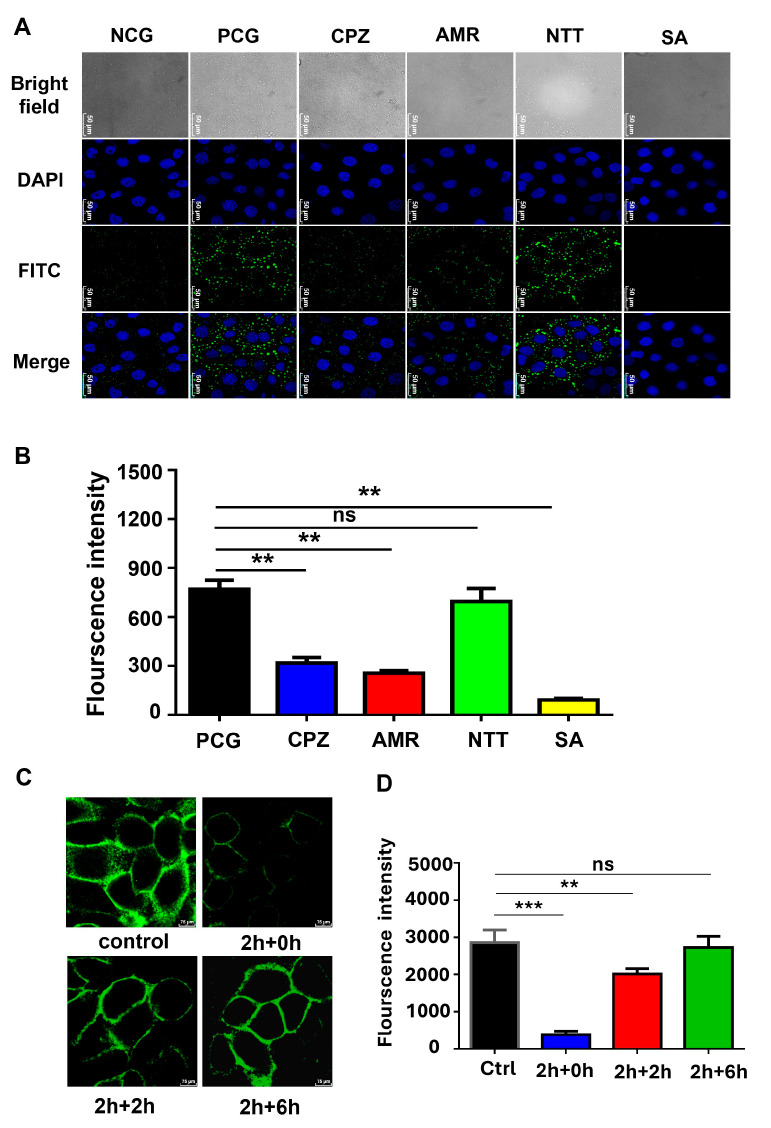
Insulin uptake mechanisms and pathways of Caco-2 monolayers. (**A**) CLSM observation to block endocytosis of FITC–insulin/NCT NP uptake by Caco-2 cells. Cell nuclei were stained with DAPI. Scale bar, 50 μm. (**B**) Quantitative analysis of fluorescence intensity of endocytosis block in Caco-2 cell uptake of FITC–insulin/NCT NPs. Total intensity of green fluorescence in each group of CLSM images was analyzed and calculated through Image J software. (**C**) CLSM images of Caco-2 monolayer stained for TJ protein (occludin) after incubation with insulin/CNT NPs at different times. Scale bar, 75 μm (**D**) Total fluorescence intensity (green) of TJ at different time periods. All values are presented as means ± SD (*n* = 3). ns, not significant (*p* > 0.05), ** *p* < 0.01, *** *p* < 0.001.

The specific endocytosis mechanism of the NCT NPs was further characterized in small intestinal epithelial cells. In this study, chemical endocytosis inhibitors were used to perform cellular endocytosis-blocking experiments to investigate the type of endocytosis of FITC–insulin/NCT NPs in Caco-2 cells, such as amiloride (AMR, a macropinocytosis inhibitor), nystatin (NTT, a small foveal protein and lipid raft-mediated cytosol inhibitor), chlorpromazine (CPZ, a lattice protein-mediated cytosol inhibitor), and sodium azide (SA, an energy-dependent cytosol inhibitor). As shown in Figure 6A, different cytosolic inhibitors resulted in different levels of cellular uptake of FITC–insulin/NCT NPs. The green fluorescence of intracellular FITC–insulin/NCT NPs appeared to be significantly attenuated in the three cytosolic-blocking experimental groups treated with AMR, CPZ, and SA. As shown in Figure 6B, the green fluorescence of lipid raft-mediated and foveolar protein-mediated cytosolic inhibitors was significantly diminished in the group treated with the cytosolic inhibitor AMR or CPZ compared to the positive control group (PCG) in an incubation environment without the addition of any inhibitors. The difference in green fluorescence between the inhibitor NTT group and PCG was not significant. The experimental results indicated that the energy-dependent endocytosis of FITC–insulin/NCT NPs by Caco-2 cells may be closely related to the lattice protein-mediated endocytosis and cytosolic drinking mechanism and less related to small fovea protein- and lipid raft-mediated cytosolization, which was consistent with reports in the literature on the mechanism of nanoparticle uptake by Caco-2 cells [48,49].

### 3.5. NCT NPs Regulate Small Intestinal Tissue Tight Junctions

Tight junctions (TJs) between intestinal epithelial cells are the primary barriers limiting oral insulin absorption. Among intestinal epithelial cells, TJs can be opened by their degradation or inactivation. The positive surface charge of chitosan nanoparticles initiates the opening of TJs in the intestinal epithelium, which in turn triggers a more extensive disassembly of TJs and stronger cell permeability. The mechanism of action may be related to the synergistic effect of Ca^2+^ blockade and clin-4 dephosphorylation. Several studies have shown that TMC opens the TJs between epithelial cells, thereby facilitating the transport of hydrophilic and peptide molecules through the paracellular pathway [50,51]. The integrity of the Caco-2 cell monolayer tight junction protein (ZO-1) was visualized using immunofluorescence. As shown in Figure 6C, FITC–insulin/NCT NPs exerted a significant effect on the TJs of Caco-2 cell monolayers. In the control group, the TJs on Caco-2 cells showed a tight and continuous ring-like distribution. After 2 h of intervention with FITC–insulin/NCT NPs, the TJs around the cell became blurred, localized, and even disappeared, demonstrating that NCT NPs can open TJs. After removing the FITC–insulin/NCT NPs and continuing the incubation, green fluorescently labelled TJs gradually reappeared, and the fluorescence signal became stronger as the incubation time increased. After 6 h of incubation, the TJs were essentially closed and returned to the closed state observed similarly in the control group. Combined with the quantitative analysis of green fluorescence using Image J software, the fluorescence intensity of TJs was significantly lower than that of the control group after incubation with NCT NPs for 2 h (*p* < 0.01). Although the fluorescence intensity of TJs increased after removing NCT NPs for 2 h, it differed significantly from the control (*p* < 0.05) until 6 h post-incubation. No statistically significant difference was observed between the fluorescence intensities of TJs and the control group after 6 h (*p* > 0.05). These results suggested that the TJ channels in Caco-2 cell NCT NPs could be reversibly opened, indicating a potential paracellular pathway for insulin uptake by Caco-2 cells.

### 3.6. Insulin/NCT NP Distribution In Vivo

**Figure 7:**
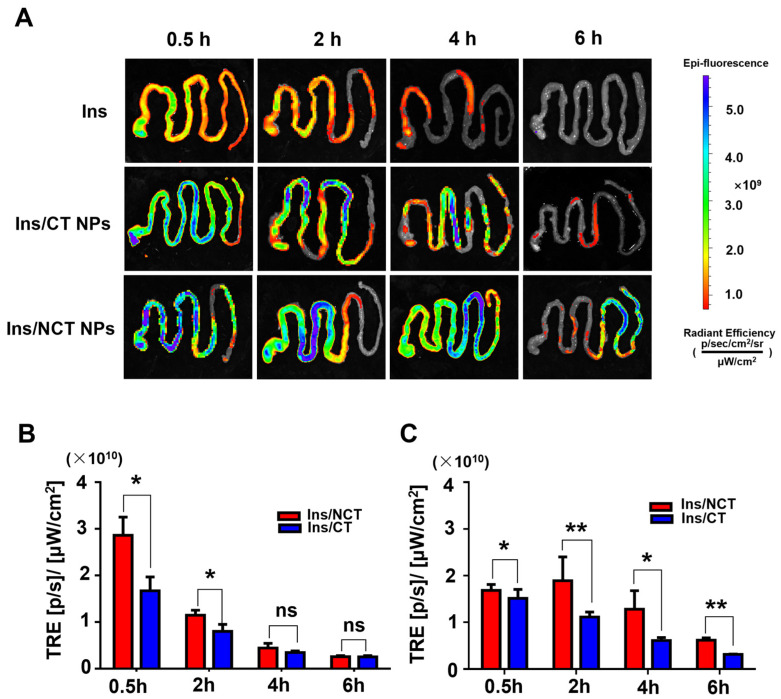
(**A**) The live imaging of the whole isolated GIT for mice following the administration of different insulin formulations in vivo. The quantification of total fluorescence in the stomach (**B**) and small intestine (**C**). All values are presented as means ± SD (*n* = 3). ns, not significant (*p* > 0.05), * *p* < 0.05. ** *p* < 0.01.

To investigate the course of insulin/CNT NPs after oral administration, Cy5.5–insulin/CT NPs were used as controls. Cy5.5–insulin, Cy5.5–insulin/CT NPs, or Cy5.5–insulin/NCT NPs were administered to mice at different time points, and the entire gastrointestinal tract (below the pylorus of the stomach to above the cecum) was observed. The whole stomach and small intestine were placed on a black plastic plate and scanned in IVIS. The Total Radiant Efficiency (TRE) of the fluorescence signals of the stomach and the small intestine was quantified. Retention and distribution in the gastrointestinal tract were also evaluated. As shown in Figure 7A, a weak fluorescent signal was visible in the gastrointestinal tract after 0.5 h of Cy5.5–insulin gavage; the fluorescent signal became weaker after 2 h, and the fluorescent signal was no longer visible after 4 h.

The fluorescent signal inside the stomach gradually decreased after the administration of Cy5.5–insulin/NCT NPs, owing to gastric emptying and the dissolution of some of the nanoparticles. Conversely, insulin/NCT NPs maintained a stronger Cy5.5 fluorescence signal in the small intestine for up to 6 h. In a preliminary experiment, it was observed that after the administration of Cy5.5–insulin/NCT NPs, occasional weak fluorescence signals were observed in the colon, suggesting that the insulin/NCT NPs were primarily solubilized and absorbed in the small intestine. The Cy5.5 fluorescence signals of insulin/NCT NPs in the stomach and intestines were stronger than those of insulin/CT NPs during the same time period. The retention time in the small intestine was at least 2 h longer than that of the insulin/CT NP group, suggesting that insulin/NCT NPs exhibit more significant mucosal adhesion permeability, which allows them to adhere and be retained in the gastrointestinal tract and prolongs the absorption time of the drug. To compare the absorption of the two nanodrug delivery systems in the gastrointestinal tract, fluorescence quantification was performed by measuring TRE values in the gastrointestinal tract. As shown in Figure 7B, the fluorescence intensity of insulin/NCT NPs in the stomach was significantly different from that of the insulin/CT NP group in the first 2 h (*p* ˂ 0.05); the fluorescence intensity was significantly stronger than that of the insulin/CT NP group in the small intestine for all 6 h (*p* ˂ 0.05); and the total fluorescence intensity in the small intestine was significantly higher than that of the stomach, which also further indicated that the insulin/NCT NPs were primarily absorbed in the small intestine. These results were consistent with the results of previous insulin release experiments in vitro.

### 3.7. Mechanism of Oral Hypoglycemic Action of Insulin/NCT NPs

**Figure 8:**
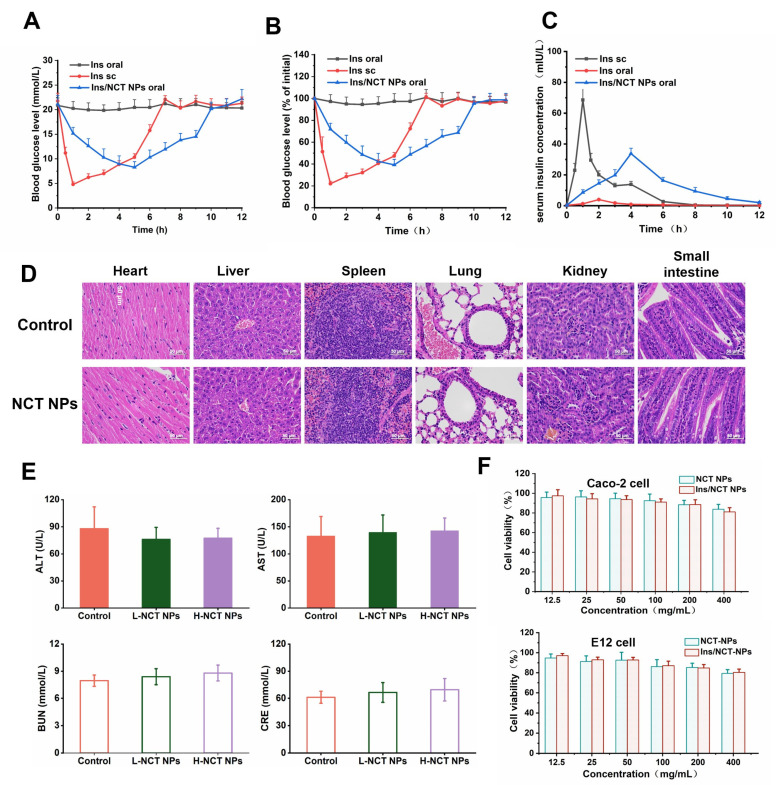
(**A**,**B**) Blood glucose level vs. time profiles of diabetic mice following administration of different insulin formulations. (**C**) Serum insulin level vs. time profiles of diabetic mice following administration of same formulations in A. (**D**) Histopathology analysis of heart, kidney, liver, and spleen tissue of mice in oral subacute toxicity study. (H&E staining; scale bar = 50 μm) (**E**) Effects of NCT NPs on mouse hepatic function and renal function. (**F**) Effect of NCT NPs and Ins/NCT NPs with distinct concentrations on viability of Caco-2 cells or E12 cells.

As shown in Figure 8A,B, the insulin subcutaneous injection group exhibited a rapid reduction in basal hyperglycemia in mice with DM, and the blood glucose level was reduced to a minimum of 22% of the basal blood glucose level at 1 h. However, after that, the blood glucose level increased rapidly. The death of individual mice was due to hypoglycemia. Because free insulin is degraded by trypsin in the gut, the hypoglycemic effect was low throughout the experiment, and the mice were essentially maintained in a hyperglycemic state. The blood glucose in the oral insulin/NCT NP group decreased more slowly than that in the subcutaneous injection group. Blood glucose decreased to the lowest level at 5 h after administration, which was approximately 39% of the initial blood glucose level, and the glucose-lowering effect was maintained for nearly 6 h. In conclusion, oral insulin/NCT NPs have a smooth and sustained effect on reducing basal hyperglycemia and are safer than subcutaneous injections.

Additionally, the pharmacokinetics of the insulin/NCT NPs were explored. As shown in Figure 8C, the serum insulin concentration peaked at 1 h in the insulin subcutaneous injection group, and C_max_ was higher than that in the other two groups. Serum insulin concentrations peaked at 4 h in the oral insulin/NCT NP group, and the rate of insulin absorption and elimination was milder compared with that in the subcutaneous injection group. The pharmacokinetic parameters of insulin are listed in Table S2. The bioavailability of oral free insulin was only 0.7%. In contrast, the relative insulin bioavailability of oral insulin/NCT NPs increased to 12.58% at the same dose, 18-fold higher than that of the free insulin group. These results suggested that insulin/NCT NPs could significantly improve insulin absorption in the gut by effectively overcoming mucus and intestinal epithelial cell barriers.

### 3.8. Safety Assessment In Vitro and In Vivo

The assessment of cytotoxicity is a critical component of drug carrier safety evaluation. In this study, the toxic effects of NCT and insulin/NCT NPs on Caco-2 and E12 cells were determined using the MTT assay. As shown in Figure 8D, the cell survival rates of the NCT NP and insulin/CT NP groups were higher than 80% within the range of 12.5–400 μg/mL. This indicated that both types of nanoparticles are safe for the cells.

Subsequently, a safety assay was performed in vivo. After 30 d of continuous feeding, no mice died, and all mice in the NCT NP group showed good growth and development, drank and ate normally, had glossy coats, moved freely, and showed no abnormalities in urination or defecation. Alanine transaminase (ALT) and aspartate transaminase (AST) levels are important indicators of liver function, whereas blood urea nitrogen (BUN) and creatinine (CRE) levels are important indicators of kidney function. The effects of orally administered NCT NPs on liver and kidney functions in mice are shown in Figure 8E. The experimental results showed no significant difference between ALT, AST, BUN, and CRE in the high- and low-dose NCT NP groups compared with the control group (*p* > 0.05). As shown in Table S3, the effect of the oral administration of NCT on other biochemical indicators in the blood of experimental mice varied. The experimental results suggested that the total protein, albumin, globulin, γ-glutamyl transpeptidase, and total bilirubin in the blood of mice in both groups orally administered with NCT NPs did not show any significant difference (*p* > 0.05) when compared with the normal control group. At the end of drug administration, the results of the H&E staining of the tissues of each group of mice are as shown in Figure 8F, and no significant histopathological changes were observed in the vital organs and small intestinal tissues in vivo. As shown in the H&E-stained sections, the myocardial fibres of the mice in each group were tightly aligned and regular, and no degeneration or necrosis of the cardiomyocytes or changes in the interstitial vessels were observed. The hematocytes in the liver tissue of mice were closely aligned with intact lobules, and the nuclei of the cytosolic cells were well-defined and of normal size. In contrast, the red and white medulla oblongata were visible in the spleens of mice in all groups with normal amounts, and no inflammation or necrosis was observed. The lung tissues of the mice in each group had reticular structures with a normal number of alveoli. The kidney structure of the mice in each group was well-defined, the brush borders of the renal tubules were complete, and no inflammatory cell infiltration was observed in the interstitium of the kidney. The gut mucosa of the mice in each group was morphologically and structurally intact, the intestinal villi were arranged in a neat manner, and the crypts were shallow with distinct limits. The experimental results showed that NCT NPs did not cause significant pathological changes in crucial organs and suggested good histocompatibility.

## 4. Conclusions

To improve the quality of life for patients with DM, oral insulin delivery offers a promising strategy by more closely mimicking endogenous insulin secretion than that of subcutaneous injections. In this study, a novel thiolated trimethyl chitosan-grafted β-cyclodextrin macromolecule (NCT) was proposed and constructed. NCT retained the cationic polymer characteristics of TMC with efficient biocompatibility and degradability while exhibiting good water solubility and mucosal adhesion. The grafting of NAC and CMCD imparted NCT with the mucosal adhesion and permeability of thiolated polymers along with the highly efficient and stable drug-carrying properties of CMCD, creating a novel macromolecule with diverse physiological activities. Moreover, the studies demonstrate in vitro and in vivo that NCT NPs effectively overcome multiple barriers to oral absorption, including pH variation, protease degradation, and mucus penetration, elucidating the mechanism of insulin/NCT NP uptake in intestinal epithelial cells. Collectively, insulin/NCT NPs significantly increased the bioavailability of insulin and effectively reduced basal glucose levels in mice with DM.

## Data Availability

The original contributions presented in this study are included in the article/Supplementary Material. Further inquiries can be directed to the corresponding authors.

## Supplementary Materials

The following supporting information can be downloaded at https://www.mdpi.com/article/10.3390/pharmaceutics18010097/s1, Table S1: Particle size, polydispersity index, and zeta potential of different nanoparticles; Table S2: Pharmacokinetic parameters from the profiles of peripheral serum insulin concentration versus time; Table S3: Effect of NCT NPs on mouse biochemical values.
