# Supplementary material for: Efficient Oral Insulin Delivery Through Thiolated Trimethyl Chitosan-Grafted β-Cyclodextrin Nanoparticles
Source: Pharmaceutics. 2026 Jan 12;18(1):97. doi: 10.3390/pharmaceutics18010097 (PMC12845214; doi:10.3390/pharmaceutics18010097)
Supplement: Supplementary file 1 [file pharmaceutics-18-00097-s001.zip › pharmaceutics-4014139-supplementary.pdf]

pharmaceutics

Supplementary materials

Efficient Oral Insulin Delivery through Thiolated Trimethyl Chitosan-grafted beta-cyclodextrin Nanoparticles

Lizhen Yu [1,2,†], Fengge Wang [2,†], Shuyun Bao [2], Yue Zhang [2], Xuebin Shen [2], Desheng Wang [2], Zhisheng Liu [2], Xinyi Liu [2], Lihua Li [2,*] and Renmin Gong [1,*]

1 Anhui Provincial Key Laboratory for Conservation and Exploitation of Biological Resources, School of Life Science, Anhui Normal University, Wuhu 241000, China; yulizhen@wnmc.edu.cn
2 School of Pharmacy, Wannan Medical College, Wuhu 241000, China; 20090016@wnmc.edu.cn (F.W.); shuyunbao@wnmc.edu.cn (S.B.); 20050003@wnmc.edu.cn (Y.Z.); sxbchn@wnmc.edu.cn (X.S.)
* Correspondence: LLH@wnmc.edu.cn (L.L.); rinmingong@ahnu.edu.cn (R.G.)
† These authors contributed equally to this work.

Table S1. Particle size, polydispersity index and Zeta potential of different nanoparticles (n=3)

Formulation	Mean size (nm)	PDI	Zeta potential (mV)
CT NPs	242.45±7.32	0.105±0.0065	+26.8±4.09
NCT NPs	224.86±7.22	0.147±0.0046	+25.5±3.90

Table S2. Pharmacokinetic parameters from the profiles of peripheral serum insulin concentration versus time (n=6)

Formulation	Insulin s.c.	Insulin oral	Insulin/NCT NPs oral
Dose (IU/ kg)	5	50	50
AUC (mIU*h /L)	113.8	8	143.2
C_{max} (mIU/ L)	68.4	3.7	33
T_{max} (h)	1	2	4
F (%)	100	0.7	12.58

Table S3. Effect of NCT NPs on mice biochemical values (n=6)

Groups	TP (g/L)	ALB (g/L)	GLB (g/L)	GGT (U/L)	TBIL (μmol/L)
Control	61.10±1.83	32.94±1.40	27.36±1.10	18.56±4.38	2.87±0.97
L-NCT NPs	60.50±3.01	31.48±1.21	31.48±1.21	16.67±2.92	2.86±0.90
H-NCT NPs	59.72±4.21	31.13±2.74	30.13±2.74	17.65±3.36	3.23±0.92

 https://doi.org/10.3390/pharmaceutics18010097
